# Adsorption Thermodynamics and Kinetics of Resin for Metal Impurities in Bis(2-hydroxyethyl) Terephthalate

**DOI:** 10.3390/polym12122866

**Published:** 2020-11-30

**Authors:** Noor Fatima, Qi Zhang, Ruru Chen, Dongxia Yan, Qing Zhou, Xingmei Lu, Jiayu Xin

**Affiliations:** 1 School of Chemical Engineering, University of Chinese Academy of Sciences, Beijing 100049, China; noor_fatima288@yahoo.com (N.F.); pzhang19@ipe.ac.cn (Q.Z.); xmlu@ipe.ac.cn (X.L.); 2 Beijing Key Laboratory of Ionic Liquids Clean Process, CAS Key Laboratory of Green Process and Engineering, State Key Laboratory of Multiphase Complex Systems, Institute of Process Engineering, Chinese Academy of Sciences, Beijing 100190, China; rthertme@163.com (R.C.); dxyan@ipe.ac.cn (D.Y.); qzhou@ipe.ac.cn (Q.Z.); 3 Innovation Academy for Green Manufacture, Institute of Process Engineering, Chinese Academy of Sciences, Beijing 100190, China; 4 Sino Danish College, University of Chinese Academy of Sciences, Beijing 100049, China

**Keywords:** Amberlite IR-120 resin, polyethylene terephthalate, bis(2-hydroxyethyl) terephthalate

## Abstract

Adsorption of heavy metals from degraded of Polyethylene terephthalate (PET) products by strong cation exchange resin AmberliteIR-120 under optimized conditions toward the selectivity removal of metals are in the following order: Al^3+^ > Zn^2+^ > Mg^2+^ > Fe^2+^ > Ni^2+^. Therefore, kinetic and adsorption isotherm models were applied for fitting experimental data. Comparatively, adsorption isotherm study revealed that Langmuir isotherm model better fits adsorption on surface of resin over than the Freundlich model. In summary, AmberliteIR-120 strong acid cation exchange resin can be used as an efficient adsorbent for heavy metals removal from depolymerized products bis(2-hydroxyethyl) terephthalate.

## 1. Introduction

Plastics are becoming popular as an industrial candidate and fundamental objects in daily consumer’s life; thus plastic material possess some unique properties which can make them useable and can be recycled by any route, and most importantly is the process is economically inexpensive [1]. Considering the production rate, plastics waste is continuously on the increased, and the conversion of these materials into a useable chemical product by recycling approach help to mitigate the accumulation in the environment. Petcore recently released that European collected post-sorting polyethylene terephthalate (PET) reached 1.13 million tons in 2007, and up about 20% from 2006. In 2007, about 40% of PET bottles were collected and consumed for recycling purposes [2]. EPA (the United States Environmental Protection Agency) reported in 2017, plastics generation was 35.4 million tons in the US, while 26.8 million tons of landfills were generated; consequently, littered our environment in a large quantity [3].

Therefore, as plastics waste remain in the environment, they deteriorate and leached out heavy metal ions into the landfill, which derived from different polymerization catalysts, additives, and recycling processes. Heavy metals persistence led to an adverse effect on population because of toxicity at lower-level caused many health problems [4,5] their non-degradability and drainage from different industries, such as metal plating facilities, fertilizers, batteries, paper industry, and tanneries would directly or indirectly discharge heavy metals into the environment [6]. Taking into account the threat of metal non-degradability, the removal of such metals is benign for the organism. They do not metabolize by the body easily; thus, enters into the human body and move into the soft tissue by different means, including food, air, contact with pesticides, pharmaceuticals, toxic chemicals, etc. [7,8]. Because of these considerations, the elimination of such toxic substances from the aquatic and soil media is imperative.

Recently, environmental demand for the chemical degradation or recycling of PET is moving towards sustainability. To this end, the use of environmentally friendly degradation methods would reduce emissions to a greater extent and save energy [9]. Chemical degradation of PET receives different products depending on the use of a solvent such as ethanolamine in aminolysis, methanol in alcoholysis [10], water in hydrolysis, and ethylene glycol in glycolysis [11,12,13]. Taking into account the high efficiency catalysis, high monomer yield, and the formation of new raw materials, glycolysis can use mild conditions in the recovery process and obtain pure monomers with higher yields [14]. Nevertheless, the obtained degraded monomer of PET has various applications as a building block in the synthesis of other valuable and economical polymeric material, including but not limited unsaturated polyester, polyurethane foams, plasticizer, textile dyes, softener, low temperature curable resins, polyurethane coating, alkyd resins, and co-polyesters. This performance is foreseen to be a beneficial, economical, and feasible choice when using the raw form of recycled monomer [15,16,17,18]. Due to the presence of the specific functional group, recycled PET products used for the removal of metal ions varied from different media to solve several problems related to soil pollution, since they appeared as a significant candidate for metal ions removal [19,20,21]. In contrast, virgin PET cannot be utilized for this purpose because it produces new waste that could be contributed to the global plastic deposition problem.

In addition, there are different physical and chemical methods for heavy metal ion uptake, such as chemical precipitation, ion exchange, coagulation, reverse osmosis, ultra-filtration, electrodialysis [22,23,24], membrane filtration [25], and flocculation [26]. These methods possess their limitation due to the nature of metals, concentration, and complexity of the solution. For example, a large amount of toxic sludge would be generated during the precipitation process, and the selectivity to specific metal ions is low [27]. Among them, the most valuable, considerable, and straightforward method is ion exchange. Therefore, ion exchange is the most commonly used in the ion exchangers. And are available in natural or synthetic resin, reusability, regeneration, and can efficiently remove various metals even at low concentrations [28]. Interestingly, an ion exchange resin is considered as the effective metal ion removal from water and industrial wastewater sources [29]. The ion exchange resins are insoluble species in organic and aqueous solution, bearing charged functional group carrying mobile ion by covalent interaction and cross-linked polymer matrix structure [30], which provides the more surface area to enhance the removal efficiency [31].

Numerous literatures have been reported previously with many resins. However, most of the literatures published are based on synthetic resin. According to reports, AmberliteIR-120 has been successfully used to remove Al, Fe, Mn from industrial wastewater [30], besides, this type of resin can also remove Al, Ni, Cr from anodic plating wastewater [32].

Herein, we investigated and studied the potential of Amberlite-IR-120 resin for the metal uptake of Al(III), Fe(II), Ni(II), Zn(II) and Mg(II) from the degraded product of PET. Similarly, the study is aimed to examine the effect of different parameters such as contact time, temperature, and resin dosage on the removal efficiency of metal ions from the degraded product of PET. Moreover, the kinetic model and isothermal adsorption model were used to fit experimental equilibrium data; the phenomenon reflected by the corresponding model was also described.

## 2. Materials and Methods

All chemical reagents were purchased without additional treatment; PET was collected and processed through the following steps: water washing, drying, and grinding into granules or powder form; sulfuric acid (H_2_SO_4_, purity ≥ 99%, Sigma, Beijing, China), nitric acid (HNO_3_, Purity ≥ 99%, Sigma, Beijing, China), ethylene glycol (C_2_H_6_O_2_, purity ≥ 99%, Sigma, Beijing, China) and zinc acetate (Zn (CH_3_COO)_2_, purity ≥ 99%, Sigma, Beijing, China) are all analytically pure; metal standard solution (Fe, Ni, Mg, Al, Zn, purity ≥ 99%, Sigma, Beijing, China); AmberliteIR-120 resin (Shanghai McLean Biochemical Co., Ltd., Shanghai, China).The physical and chemical properties of the resin bearing H^+^ in an ionic form; functional group, sulfonic acid; maximum operating temperature, 120 °C; polymer matrix, styrenedivinylbenzene.

C-MAG HA-7 IKA magnetic stirrer (German Aika, Deutschland, Germany) was used to keep temperature constant. To remove the excessive amount of water, RE2000 rotary evaporator (Shanghai Yarong Biochemical Instrument Factory) and a DHG9053A blast drying oven (Shanghai Yiheng Scientific Instrument Co., Ltd., Shanghai, China) were used. Furthermore, ICPE-9000 Inductively Coupled Plasma Emission Spectroscopy (Shimadzu Corporation, Tokyo, Japan.) technique were employed to analyze the metal concentration. In whole experimental setup, ultra-pure water (Milli-Q Direct 8 Ultrapure Water Machine-Merck Millipore Corporation, Darmstadt, Germany) were equally used. The surface characterization of resin has been performed by SEM (Scanning Electronic Microscopy, Beijing, China) technique.

**Figure 1:**
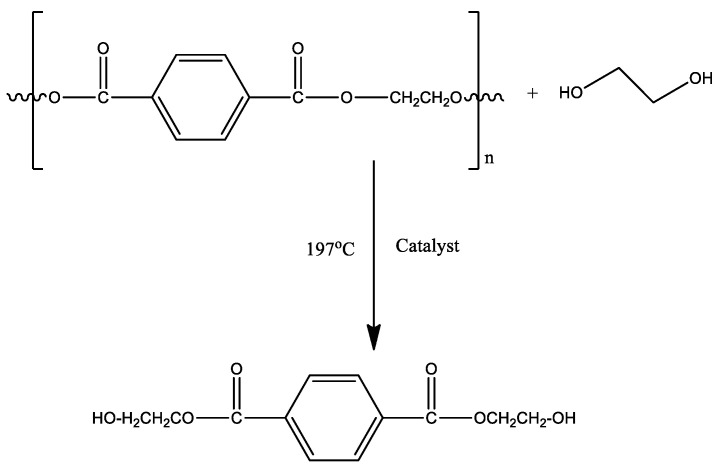
Chemical depolymerization of (PET)*n* into product bis(2-hydroxyethyl) terephthalate BHET.

In addition, depolymerization of PET is carried out according to the previous reported method [33] and modification was made accordingly. In brief, firstly, 100 g of PET and 400mL of EG solvent are mixed and added into a three-necked flask equipped with a thermometer and a reflux condenser operated at temperature 197 °C, then small amount of catalyst for catalytic degradation was added, the entire experimental process is performed at atmospheric pressure; when the depolymerization process is completed, a filter was used to remove insoluble dimer and unreacted PET, and then add excess ultrapure water to the filtrate dissolve the monomer and place it at 4 °C for crystallization. Finally, filtrate was dried to obtain BHET monomer according to the scheme depicted in Figure 1.

In a typical experiment, 1 g of BHET monomer was mixed with AmberliteIR-120 sorbent of (0.02 g) in 40 mL volume in round bottom flask equipped with a magnetic stirrer at designated time. The concentration of the solution prepared (20–500 ppm). Phase separation was performed by filtration and metal ion concentration was determined by ICP. The different experiments were conducted to optimize the metal uptake parameters according to the sorbent efficiency of ion exchange of metals on the resin, including reaction temperature (293.5–348.5 K), resin dosage (0.005–0.2 g), and reaction time (0.5–120 min). The stirring speed was kept at 150 rpm to maintain resin particle in solution.

Sorption capacity of each metal was calculated by using the following equation [34]:

(1) $$q_{t}=\frac{\left(Co-Ct\right)V}{m}$$

where *q_t_* was used to represent the amount of adsorbed metal per gram; *Co* relates the initial concentration of metal in the solution (mgL^−1^); *Ct* shows the equilibrium concentration of metal in the solution (mgL^−1^) after time period at time *t*; the treated solution can be represented at volume *V* in (mL), and mass of sorbent *m* in treated solution (g). However, given formula used to calculate the removal percentage of metal (*R*%):

(2) $$R\left(\%\right)=\frac{\left(Co-Ct\right)}{Co}\times 100$$

## 3. Results and Discussion

The BHET monomer was studied with ^1^HNMR, FTIR and ^13^CNMR, and DSC thermogram (the brief description was provided in supporting information as Figure S1). Further, the BHET monomer has been characterized by ICP technique. In brief, a fixed weight of BHET was digested by the help of Di-acid solution (H_2_SO_4_ and HNO_3_), and then three parallel solutions were characterized under standard metal solution. To this end, the average value was considered. Analysis of digested BHET in di-acid (HNO_3_ and H_2_SO_4_) (1:4) mixture showed different metals are present in BHET. BHET found with variety of metals under different ranges in (ppm). Upon evaluation of the concentration, five metals with highest concentration were chosen for removal study. The concentrations of metals were selected above (>50 ppm) for removal content from BHET. Therefore, the concentration of Mg metal content was identified with higher concentration 349 ppm (14.36 mmol/L), while the rest are present in the following ratio Ni 343 ppm (5.85 mmol/L), Al 185 ppm (6.86 mmol/L), Fe 122 ppm (2.18 mmol/L), and Zn 109 ppm (1.67 mmol/L). The order of metal presence (in mg metal L^−1^) falls into the following order of matrix: Mg^2+^ > Ni^2+^ > Al^3+^ > Fe^2+^ > Zn^2+^. On the other hand, the concentration in mmol metal L^−1^ follows the order: Mg^2+^ > Al^3+^ > Ni^2+^ > Fe^2+^ > Zn^2+^.

Resin selectivity performance was carried out with various commercially available resins including strong and weak cation resins. Favorability of best resin among different resins was achieved by comparing the removal percentage of metals. The strict comparison is difficult since maximum removal percentage were not obtained under similar experimental conditions. However, these data are sufficient to show thatAmberliteIR-120 sorbent have comparable removal capacity than conventional sorbents. The main advantage of the reported sorbent is the fast kinetics of sorption. The results for the best resin performance are shown Table S1.

### 3.1. Metal Uptake Parameter Optimizations

The effect of resin dosage on metal removal was studied and analyze with special fixed amount of BHET powder, and results were explained in details. The varied resin amount was treated with BHET in the batch experiment at the constant speed of 150 rpm and a 318 K temperature under a fume hood. As the adsorbent amount is a significant parameter used to study the maximum metal adsorption; thus provides an insight into the resin dosage. In view of this consideration, designing a different experimental setup with different amounts of resins while keeping other parameters constant in order to establish the relationship with the optimum amount of resin with higher adsorption capacity is imperative.

**Figure 2:**
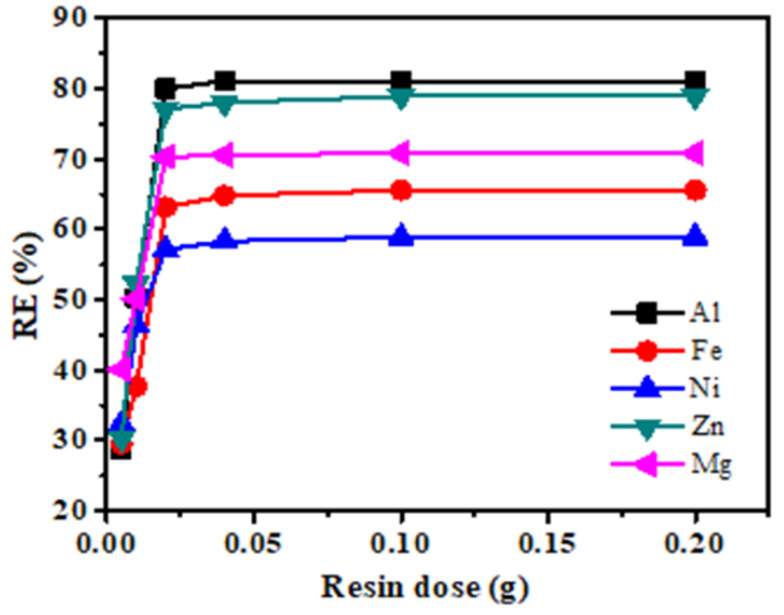
Effect of resin dosage on the ion exchange of Al(III), Fe(II), Zn(II), Mg(II) and Ni(II) using the AmberliteIR-120 (temperature: 318 K; stirring speed: 150 rpm; contact time, 15 min).

As depicted in Figure 2, different amount of resin in the following range from (0.005, 0.01, 0.02, 0.04, 0.1 and 0.2 gL^−1^) has been used. The maximum removal efficiency of metals was obtained with 0.02 gL^−1^ of resin contact, as shown in Figure 2, follows the order Al^3+^ > Zn^2+^ > Mg^2+^ > Fe^2+^ > Ni^2+^ respectively. This may be as result of lower resin amount, hence the sites of resins might approach the saturation stage; an abundant amount of heavy metals ion was present in the solution, and lower removal efficiency was reported. However, on the other hand, by increasing the resin dosage, maximum removal efficiency is interchanged with the possible number of the exchangeable site available [32]. In contrast, the overloading of the resin dosage into solution attributed to the lower sorption uptake since approximately all the metals ions were exchanged at the available site very quickly at a lower concentration.

**Figure 3:**
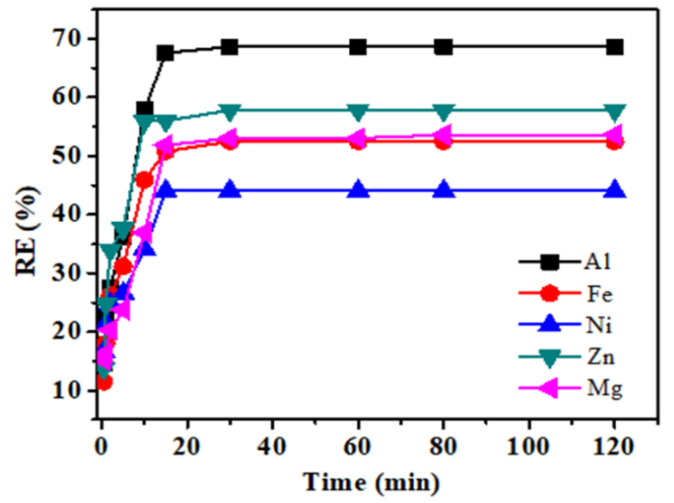
Effect of time on the uptake of metals Al(III), Fe(II), Zn(II), Mg(II) and Ni(II) using AmberliteIR-120 (temperature, 318 K; sorbent dose, 0.02 g; stirring speed, 150 rpm).

Retention time is a significant factor in defining the efficiency of any adsorbent [35]. The removal of metal percentage studied was done by varying the contact time. The time effect was collected in a batch experiment of 1 g of BHET with a 40 mL volume of solution, given the amount of resin with the constant speed rate of 150 rpm. The different experiments were established with the deal of varying contact time 1, 2, 5, 10, 15, 30, 60, 80, and 120 min, respectively. Metal concentration showed their behavior differently at each point. It can be seen from Figure 3 that the initial 0–15 min belongs to the rapid adsorption stage, and then with the extension of the adsorption time. The removal rate of metal ions increases slowly and finally reached the adsorption equilibrium. Therefore, adsorption behavior exhibited at each time point is not the same, and the curves in the Figure 3 are the monotonous and steady rise and eventually reach saturation, which shows that the metal ions in the solution are likely to be covered by a monolayer on the surface of the resin.

Metals adsorption reached high plateau adsorption at some points, but maximum adsorption achieved at 15 min after which the metals adsorption fall under equilibrium state. The metal adsorption showed that minimum contact time is required for the resin to adsorb metals, as shown in Figure 3.

**Figure 4:**
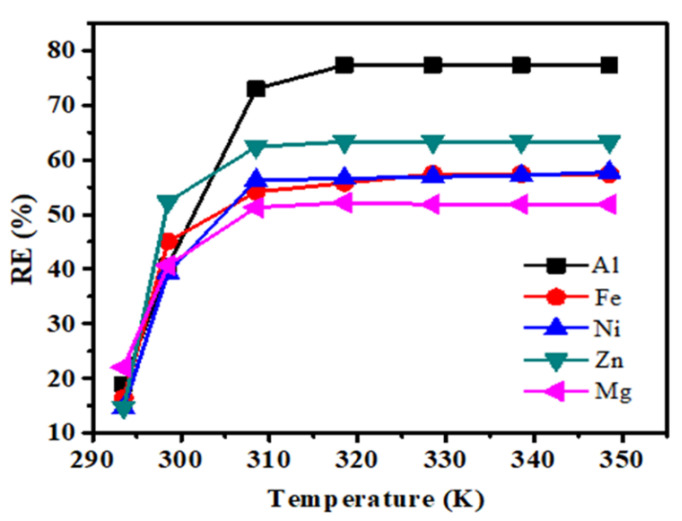
Effect of temperature on metal uptake of Al(III), Fe(II), Zn(II), Mg(II) and Ni(II), using AmberliteIR-120 (time contact, 15 min; sorbent dose, 0.02 g; stirring speed, 150 rpm).

Temperature is important parameter to establish the reaction reactivity at a specific point. For the metal adsorption, BHET powder was prepared in batch experiment conducted at different temperature ranges: 293 K, 298 K, 308 K, 318 K, 328 K, 338 K, and 348 K with the constant speed limit of 150 rpm for 15 min. As depicted in Figure 4**,** as the temperature increases, the removal efficiency (RE) of each metal ion in the solution also gradually increases. The effects of the further increase on the removal rate of metal ions will no longer change. This implies that the rise in temperature is conducive to the adsorption process. The adsorption process of AmberliteIR-120 cation exchange resin for each metal ion in solution is an endothermic process. Gradual increase in temperature significantly increases the diffusion rate and mass transfer rate between metal ions in the solution, thus enhances the chances of contact among metal ions and the interior side of the resin, which specifically increase the removal rate. To the end, as the temperature rises, the trend of the metal ion removal rate determines that 318 K is the optimal temperature.

**Figure 5:**
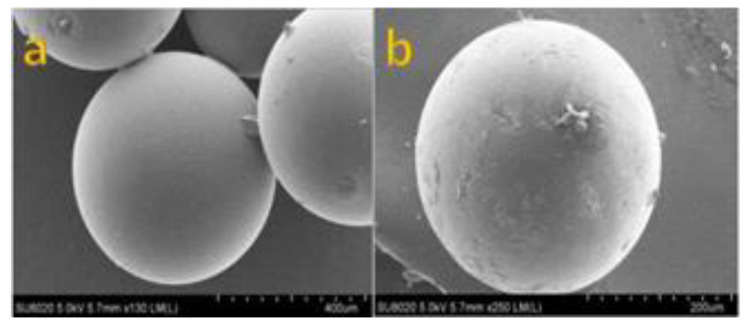
SEM images of resin; (**a**) Amberlite IR-120 before reaction with the magnification of 400 µm (**b**) resin after reaction at 318 K, 150 rpm, with the magnification of 200 µm.

The surface morphology of the resin AmberliteIR-120 was characterized by SEM for analysis before and after the reaction conditions, respectively. The surface of resin in Figure 5 clearly shows the difference, before and after the resin metal adsorption with the magnification power of 200 µm and 400 µm. Similarly, Figure 5b shows that the surface of resin becomes rough and some beads have generated on its surface indicating the metal has been adsorbed on the resin surface. This collective analysis shows that after the reaction, the surface morphology of resin has been changed due to metal adsorption indicating the successful adsorption.

Precision of metals before and after treatment of BHET has been estimated and the results were listed in Table S2. As seen from Table S2, the removal percentage of Al, Fe, Ni, Zn and Mg from BHET were 81%, 66%, 50%, 79%, and 71%, respectively. These overall values concluded that the almost all heavy metals can be adsorbed by resin AmberliteIR-120 from BHET.

### 3.2. Ion Exchange Isotherm

Adsorption behavior of solutes on the resin is more precisely studied through the adsorption isotherm [36]. Mathematical expressions of sorption isotherm are used to draw an elaborate model between liquid and solid phases [37]. Sorption data can be explained by two isotherm models; Langmuir and Freundlich isotherm respectively. Langmuir model governed the maximum adsorption capacity of the monolayer coverage of the metal on the resin surface [38]. Freundlich model used to elaborate on the relationship between metal ion concentrations in the solid phase at equilibrated state with metal ion concentration in the aqueous phase. Frequently, this model predicts the surface heterogeneity and the exponential distribution of active regions and their energies. The Langmuir isotherm principle states that the formation of a single monolayer on an adsorbent demonstrates the equivalent distribution of metal ion between liquid and solid phases, without the further formation of sorbate layer on the outer surface [39]. The Langmuir expression is represented by [40]:

(3) $$\frac{Ce}{q_{e}}=\frac{Ce}{Qo}+\frac{1}{Qo\;b}$$

where *Ce* is the equilibrium concentration (mg L^−1^) in solution, *q_e_* is the solid phase sorbate concentration at equilibrium (mg g^−1^). The constant *Q_o_* gives the theoretical monolayer adsorption capacity (mg g^−1^), and *b* is related to the energy of adsorption (L mg^−1^), and it should be dependent on temperature and vary with it. Values of *b* and *Q_o_* are calculated from the intercept and slope of the plot *Ce*/*q_e_* vs. *Ce*. This should provide a concise and precise description of the experimental results, their interpretation as well as the experimental conclusions that can be drawn.

The parameter *b*, *Q_o_* computed by the Langmuir isotherm and statistical values are shown in Table 1. The Langmuir isotherm model precisely describes the sorption data with correlation coefficient values (0.993–0.997). The value of (*b*) parameter predicts the higher affinity of metal to the sorbent surface with AmberkiteIR-120. By examining the parameter *Q_o_*, sorption phenomena related to the charge density of cation, as the diameter of the cation is higher, minimum adsorption was observed. Accordingly, the statistical values in Table 1 of Al^+3^ (*Q_o_* = 90.9) have a minimum diameter (0.53 Å) hence, shows higher sorption over other metals. While the other metals with the same charge Ni^2+^, Mg^2+^, Fe^2+^ and Zn^2+^ have similar charges thus showed minimum uptake capacity (Ni^2+^, Fe^2+^ = 0.70 Å, Mg^2+^ = 0.72 and Zn = 0.74 Å) [41]. The Freundlich isotherm model is expressed as [42]:

(4) $$\log q_{c}=\log k_{f}+\left(1/n\right)\log Ce$$

Freundlich constant is represented by *kf* in the above equation associated with the sorption capacity of the resin (mg g^−1^). Higher values of *kf* indicate a higher affinity for metals. The intensity of sorption is related to constant 1/*n* and shows that the affinity of the sorbate to be sorbed. Numerical values of constant *kf* and *n* were calculated from the plot of log *q_e_* and log *C_e_*, as shown in Table 2. Therefore, the value of *n* is best indicative of sorption in Freundlich model [43,44]. The slope of 1/*n*< 1 is a measure of adsorption intensity or surface heterogeneity. The numerical values of 1/*n* show it slightly suppressed at lower concentration and does not predict any saturation of the metals on ion-exchange resin.

### 3.3. Ion Exchange Kinetic

Metals such as Al, Fe, Ni, Zn, and Mg uptake were represented by using the conventional equation and to draw the plot log(*q_e_* − *q_t_*) versus time “*t*” in first order kinetic and *t*/*q_t_* plotted against time “*t*” in second-order kinetics to have a better insight into the kinetics of adsorption. For this purpose, *R*^2^ values were calculated in both models and kinetic of adsorption was calculated to know how the model is adequately fitted. The calculated values of *q_e_* in pseudo-second-order kinetic better explained the equilibrium capacity than the pseudo-first-order kinetics. Besides, the computed equilibrium capacity (*q_e_*, calc.) by the pseudo-second-order kinetic model proves better fitting of experimental equilibrium capacity (*q_e_*, exp.) compared to the pseudo first-order kinetic model.

According to Wolowicz and Hubicki, only in few cases, the pseudo first-order kinetic model is better fits data than PSOR [45]. PFOR kinetics of Largergren is usually represented by the expression:

(5) $$\frac{dq_{t}}{dt}=k_{1}\left(q_{e}-q_{t}\right)$$

where *q_e_* and *q_t_* related with the sorption capacity at equilibrium and at time *t*, respectively (mg g ^−1^) and *k*_1_ is the rate constant of PSOR sorption (L min^−1^). However, after integration and applying boundary conditions *t* = 0 to *t* = *t* and *q_t_* = 0 to *q_t_* = *q_t_*, the integrated form of Equation (3) becomes:

(6) $$\log\left(q_{e}-q_{t}\right)=\log\left(q_{e}\right)-\left(\frac{k_{1}}{2.303}\right)t$$

If rate order is considered second order, then the PSOR chemisorption’s kinetic rate equation is expressed as: [46,47]

(7) $$\frac{dq_{t}}{dt}=k{\left(q_{e}-q_{t}\right)}^{2}$$

where *q_e_* and *q_t_* represented the sorption capacity at equilibrium and at time *t*, (mgg^−1^) and *k* is the rate constant of pseudo-second-order sorption (g mg^−1^ min^−1^). By applying boundary conditions *t* = 0 to *t* = *t* and *q_t_* = 0 to *q_t_* = *q_t_*, the integrated form of Equation (5) expressed as:

(8) $$\frac{1}{\left(q_{e}-q_{t}\right)}=\frac{1}{q_{e}}+kt$$

This equation is the integrated rate law for a pseudo-second order reaction. Where *q_e_* is the amount of metal ion sorbed at equilibrium (mg g^−1^) and *k*_2_ is the equilibrium rate constant of pseudo-second order sorption (g mg min^−1^). Equation (6) can be rearranged to obtain a linear form:

(9) $$\frac{t}{q_{t}}=\frac{1}{k_{2}{q_{e}}^{2}}+(\frac{1}{q_{e}})t$$

where the equilibrium sorption forward *k*_1_ (Lmin^−1^) and backward *k*_2_ (g mg^−1^ min^−1^) rate constants were calculated from the plots of their respective data were estimated based on the intercept and slope of their fitted model, *q_e_* and *q_t_* are the sorption capacity at equilibrium and at time *t*, respectively (mg g^−1^).

The plot of log(*q_e_* − *q_t_*) and *t*/*q_t_* versus time “*t*” and R^2^ values in both pseudo-first-order and pseudo-second-order kinetics are presented in Table 3. A comparison of these values predicts the best fitting modelling of the pseudo second-order than first-order kinetics. It also showed that experimental values agreed well with calculated values. All these parameters in pseudo second-order kinetics assumed that adsorption studied fits well with pseudo-second-order indicating that the rate-limiting step might be chemical sorption relating adsorption activity by valence forces through sharing or exchange of electrons between sorbent and sorbate [48].

### 3.4. Application of Purified BHET

**Figure 6:**
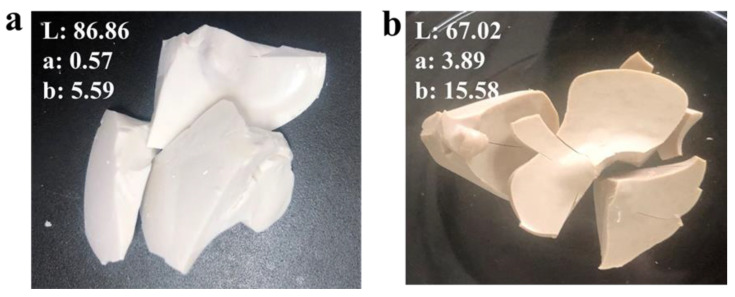
Digital images of (**a**) the rPET with purified BHET and (**b**) the rPET with unpurified BHET.

PET depolymerized into monomer BHET containing an excessive amount of heavy metals. In this work, heavy metals have been removed from monomer BHET by ion exchange resin, as their presence is considered as pollutants. If metals persist consistently into a recycled monomer it would subject to problems during the repolymerization of PET (rPET). Therefore, it is necessary to use the purified monomer to obtain a high yield PET [49]. It is believed that the presence of heavy metals contaminates can lead in the form of color impurity, which may result in lowering the quality of repolymerized PET. In this regard, the purified BHET and unpurified BHET has been used as a precursor for rPET through the mature procedure. The digital images for changes in the color of rPET were shown in Figure 6. The selected color space was the chromatic model *L*, *a*, *b* or CIE Lab (spherical color space), where *L* stands for the luminance, if *L* = 0 shows dark and if *L* = 100 indicates clarity or lightness. The *a*, *b* pair represents the chromaticity coordinate, if *a*> 0 is considered red, *a* < 0 represents green, and if *b* > 0 is yellow, and *b* < 0 is blue. The *L*, *a*, *b* coordinate values were obtained and compared with before and after resin treatment. Upon treatment with purified rPET showed (*L* = 86.86), (*a* = 0.57), and (*b* = 5.59); with unpurified treatment indicate (*L* = 67.02), (*a* = 3.89), and (*b* = 15.58). The comparison clearly identifies the lightness of rPET after resin treatment and color removal from desired product. As a substance, metals presence slightly affects the appearance and performance of PET and it has been removed from BHET by Amberlite IR-120. It exhibits that the removal of heavy metal present in the BHET results in high quality of PET. Thus, the relative pure PET with high quality was obtained and metal removal effectively recovers purified monomer BHET, which can be used in various applications to repolymerized the PET plastic.

## 4. Conclusions

In the present study, removal of metals Al(III), Fe(II), Mg(II), Zn(II) and Ni(II) were subjectedwithAmberliteIR-120 from the degraded product of PET in order to make the monomer BHET metal free. Ion exchange equilibrium conditions were studied at different factors to optimize the conditions. Sorption is efficient with 0.02 g sorbent dosage, 318 K temperature and 15 min contact time. Metals followed the maximum adsorption in the order of Al^3+^ > Zn^2+^ > Mg^2+^ > Fe^2+^ > Ni^2+^.The influence of metal uptake on equilibrium data modeled with the Langmuir and Freundlich isotherm, Langmuir model better fitted the data as compared with Freundlich model with correlation regression coefficient value R^2^ ranging from (0.993–0.997). The ion exchange kinetics for metal uptake could be predicted more accurately with pseudo-second-order kinetics. From practical perspective, AmberliteIR-120H resin exhibiting excellent properties, including low cost, easy availability for maximum adsorption metal uptake from degraded product of PET.

## Figures and Tables

**Table 1:** Parameters of Langmuir isotherm for ion exchange of metals on AmberliteIR-120.

Metals	Langmuir Isotherm		*R* ^2^
	*b*	*Q_o_*	
Al	0.019	90.9	0.997
Fe	0.050	83.3	0.997
Ni	0.010	16.6	0.990
Mg	0.095	20.0	0.994
Zn	0.023	13.8	0.993

**Table 2:** Parameters of the Freundlich isotherm model for ion exchange of metal on AmberliteIR-120.

	Freundlich Isotherm		
Metal	*kf*	1/*n*	*R* ^2^
Al	0.66	0.53	0.987
Fe	0.49	0.62	0.984
Ni	0.70	0.86	0.982
Mg	0.68	0.98	0.993
Zn	0.76	0.44	0.990

**Table 3:** Kinetic model parameters for adsorption of metal ions on AmberliteIR-120.

Metals	Pseudo-First-Order			Pseudo-Second-Order		
	*k*_1_ (L min^−1^)	*q_e_* (mg g^−1^)	*R* ^2^	*k*_2_ (g mg^−1^ min^−1^)	*q_e_* (mg g^−1^)	*R* ^2^
Al	0.165	0.227	0.973	1.08 × 10^−3^	200	0.997
Fe	0.179	0.266	0.939	1.63 × 10^−3^	142	0.998
Ni	0.131	0.378	0.957	1.6 × 10^−4^	500	0.990
Zn	0.244	0.280	0.803	1.51 × 10^−3^	166	0.999
Mg	0.082	0.330	0.944	2.10 × 10^−4^	500	0.990

## Supplementary Materials

The following are available online at https://www.mdpi.com/2073-4360/12/12/2866/s1, Figure S1: The (a) ^1^HNMR and ^13^CNMR, (b) FTIR, and (c) DSC images of BHET-1 (Before recation with resin) and BHET-2 (After reaction with resin), Table S1: Comparative analysis of metals removal efficiencies (%) of different resins with of IR-120, Table S2: Concentration of metals at different stage and their recovery efficiencies.
