# Supplementary material for: Adsorption Thermodynamics and Kinetics of Resin for Metal Impurities in Bis(2-hydroxyethyl) Terephthalate
Source: Polymers (Basel). 2020 Nov 30;12(12):2866. doi: 10.3390/polym12122866 (PMC7761184; doi:10.3390/polym12122866)
Supplement: Supplementary file 1 [file polymers-12-02866-s001.pdf]

Adsorption Thermodynamics and Kinetics of Resin for Metal Impurities in Bis (2-Hydroxyethyl Terephthalate)

Fatima Noor [1,2,4], Qi Zhang [1,2,3,4], Ruru Chen [2,4], Qing Zhou [2,4], Xingmei Lu [1,2,3,4], Jiayu Xin [1,2,3,4]*

[1] School of Chemical Engineering, University of Chinese Academy of Sciences, Beijing 100049, PR China.
[2] Beijing Key Laboratory of Ionic Liquids Clean Process, CAS Key Laboratory of Green Process and Engineering, State Key Laboratory of Multiphase Complex Systems, Institute of Process Engineering, Chinese Academy of Sciences, Beijing 100190, PR China.
[3] Sino Danish College, University of Chinese Academy of Sciences, Beijing 100049, PR China.
[4] Innovation Academy for Green Manufacture, Institute of Process Engineering, Chinese Academy of Sciences, Beijing 100190, PR China.

Table S1. Comparative analysis of metals removal efficiencies (%) of different resins with of IR-120.

Resins name	Al	Fe	Ni	Zn	Mg
**IR-120**	92	98	92	90	89
**IRC-718**	70	77	65	76	69
**D001**	84	83	77	76	86
**SIR-300**	83	78	73	80	84
**CH-90**	81	80	72	79	64

Table S2. Concentration of metals at different stage and their recovery efficiencies

**Process**	**Metals**				
	Al	Fe	Ni	Zn	Mg
Metal Content					
Before BHET treatment (ppm)	185	122	343	109	349
Metal absorb by resin (ppm)	149.85	80.52	171.5	86.11	247.7
Metal remained in BHET (ppm)	35.15	41.8	171.5	23	101.21
**Efficiency of metal removal by resin (%)**	81	66	50	79	71

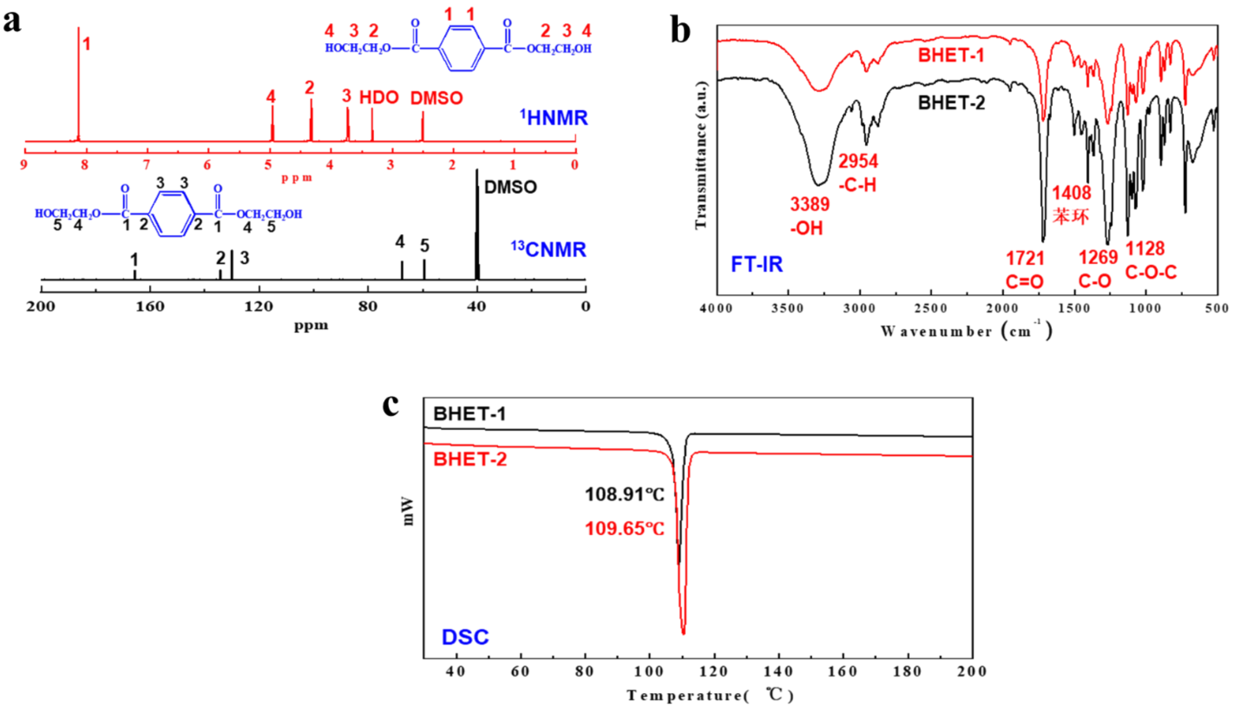

Figure S1. The (a) ^{1}HNMR and ^{13}CNMR, (b) FTIR, and (c) DSC images of BHET-1 (Before recation with resin) and BHET-2 (After reaction with resin)

BHET characterization analysis has been studied with ^{1}HNMR, FTIR, ^{13}CNMR and DSC thermogram. As shown in Figure S1(a),^{1}HNMR provided that signals at d=8.1 represent the presence of proton of the aromatic benzene ring and 3.9 ppm were characteristic pecks of methylene group. On the other hand, the triplet signal peak at d=4.2 ppm was attributed to the protons of the hydroxyl group. The signal that corresponds to the proton of the hydroxyl group was found at d=4.9 ppm. In ^{13}CNMR spectra signal peaks at 166ppm indicating the presence of carbonyl group as shown in Figure S1 (a). Signal peaks at 129.67ppm indicating methylene group and peaks at 133.83 ppm for aromatic carbons. CH_2OH and -COO-CH_2- groups are found at 67.02 and 61.26 ppm respectively.

Furthermore, as shown in Figure S1(b), FTIR spectrum of BHET provide the significant absorption signals corresponding to the OH stretching bond at 3389 cm^{-1}, the C=O stretching bond of the ester group at 1721 cm^{-1} and the C-OH peak at 1128 cm^{-1}. Moreover, DSC of degraded product of PET showed characteristic sharp endothermic peak at 110 °C in agreement with the known melting point of BHET, as displayed in Figure S1(c). From all these observations and proposed structure it can be concluded that the product from glycolysis was highly pure BHET.
